# Semi-Online Scheduling on Two Machines with *GoS* Levels and Partial Information of Processing Time

**DOI:** 10.1155/2014/576234

**Published:** 2014-02-11

**Authors:** Taibo Luo, Yinfeng Xu

**Affiliations:** ^1^Business School, Sichuan University, Chengdu 610065, China; ^2^State Key Lab for Manufacturing Systems Engineering, Xi'an 710049, China

## Abstract

This paper investigates semi-online scheduling problems on two parallel machines under a grade of service (*GoS*) provision subject to minimize the makespan. We consider three different semi-online versions with knowing the total processing time of the jobs with higher *GoS* level, knowing the total processing time of the jobs with lower *GoS* level, or knowing both in advance. Respectively, for the three semi-online versions, we develop algorithms with competitive ratios of 3/2, 20/13, and 4/3 which are shown to be optimal.

## 1. Introduction

Scheduling problem under a grade of service provision was first proposed by Hwang et al. [1]. The jobs should be assigned irrevocably to machines as soon as they arrive. Each job and machine are labeled with the *GoS* levels. A job can be processed by a particular machine if and only if the *GoS* level of the job is not less than that of the machine. In practice, the scheduling with *GoS* eligibility constraints is widely used. For more details, please refer to [1–3].

For the offline scheduling on *m* machines under a grade of service provision, Hwang et al. [1] presented an lg-lpt algorithm which has a tight bound of 5/4 for two machines and 2 − 1/(*m* − 1) for *m* (*m* ≥ 3) machines. Ji and Cheng [4] gave an fptas for this problem. However, Woeginger [5] gave two simpler fptass for the same problem. For the online version, Jiang [6] proved a lower bound of 2 for the case with two levels of *GoS* and presented an online algorithm with a competitive ratio of 2.52. Zhang et al. [7] improved the result and showed an algorithm with a competitive ratio of 1 + (*m* ^2^ − *m*)/(*m* ^2^ − *sm* + *s* ^2^) ≤ 7/3.

For *m* = 2, Park et al. [8] presented an optimal algorithm with a competitive ratio of 5/3. However, there are many papers focusing on the semi-online scheduling on two machines under *GoS* [8–13]. As the total processing time of all jobs is known, Park et al. [8] gave an optimal algorithm with a competitive ratio of 3/2. When the largest processing time of all jobs is known, Wu et al. [12] presented an optimal algorithm with a competitive ratio of $\left(\sqrt{5}+1\right)/2$. In the same paper, Wu et al. [12] gave an optimal algorithm with a competitive ratio of 3/2 when the optimal value of the instance is known. For the processing times bounded in an interval, Liu et al. [9] gave a competitive algorithm under some conditions and Zhang et al. [13] improved the result and gave an optimal algorithm for different intervals. In this paper, we focus on the semi-online scheduling problem where only partial information of the total processing time is known. The semi-online versions concerned in our paper are listed as follows.

 
*sum* · *higher*: the total processing time of all jobs with higher *GoS* level is known in advance.
 
*sum* · *lower*: the total processing time of all jobs with lower *GoS* level is known in advance.

We use *P*2 · *GoS* | *s* | *C*_max⁡_ to denote the semi-online problem with information *s*, where *s* ∈ {*sum* · *higher*, *sum* · *lower*}. Moreover, we use *P*2 · *GoS* | *s*1&*s*2 | *C*_max⁡_ to denote the semi-online problem where both information *s*1 and information *s*2 are available in advance.

Our results indicate that competitive ratios of three algorithms are better than 5/3 of the online version [8]. When knowing *sum* · *higher* in advance, the competitive ratio is the same as the optimal algorithm [8] presented for the semi-online problem where the total processing time of all jobs is known. For designing algorithm, the results indicate that knowing *sum* · *higher*&*sum* · *lower* is more useful than *sum* · *higher* or *sum* · *lower*. Moreover, knowing *sum* · *higher* is more useful than *sum* · *lower*. However, knowing *sum* · *lower* is better than knowing the largest processing time of all jobs [12].

The rest of this paper is organized as follows: in Section 2, we give some basic definitions. In Sections 3–5, we prove lower bounds and present algorithms for the three semi-online problems, respectively.

## 2. Basic Definitions

We are given two machines and a series of jobs arriving online which are to be scheduled irrevocably at the time of their arrivals. The arrival of a new job occurs only after the current job is scheduled. We denote by the set *J* = 1,…, *n* the set of all job indices arranged in the order of arrival. The *j*th arriving job is referred to as job *J*_*j*_. We denote each job by *J*_*j*_ = (*p*_*j*_, *g*_*j*_). The *GoS* assigned to job *J*_*j*_ is denoted by *g*_*j*_, which is 1 if the job must be processed only by the first machine and 2 if it may be processed by either of the two; *p*_*j*_ is the processing time of job *J*_*j*_; *p*_*j*_ and *g*_*j*_ are not known until the arrival of job *J*_*j*_. Let *T*_1_ = ∑_*g*_*j*_=1_ *p*_*j*_ and *T*_2_ = ∑_*g*_*j*_=2_ *p*_*j*_.

The scheduled can be seen as the partition of *J* into two subsets, denoted by (*S*_1_, *S*_2_), where *S*_1_ and *S*_2_ contain job indices assigned to the first and second machine, respectively. Let *t*(*S*) = Σ_*J*_*j*_∈*S*_ *p*_*j*_ for an arbitrary subset *S* of *J*. Then the maximum of *t*(*S*_1_) and *t*(*S*_2_), denoted by max⁡{*t*(*S*_1_), *t*(*S*_2_)}, is the makespan of the scheduled (*S*_1_, *S*_2_). The problem is to minimize max⁡{*t*(*S*_1_), *t*(*S*_2_)}.

The minimum makespan obtained by an optimal offline algorithm is denoted by *C*_opt_. The makespan generated by algorithm *A* is denoted by *C*_*A*_. The competitive ratio of *A* is defined to be the maximum *C*_*A*_/*C*_opt_.

## 3. Optimal Algorithm for *P*2 · *GoS* | *sum* · *higher* | *C*_max⁡_

In this section, we prove a lower bound of competitive ratio and present an optimal algorithm for the semi-online version as *T*_1_ is known in advance.

### 3.1. Lower Bound of Competitive Ratio

**Theorem 1**

Any semi-online algorithm for *P*2 · *GoS* | *sum* · *higher* | *C*_max⁡_ has a competitive ratio of at least 3/2.

**Proof**

The theorem will be proved by adversary method. Let *T*_1_ = 1 be known in advance. The first job is *J*_1_ = (1,1). *J*_1_ must be scheduled on the first machine. Then, we generate job *J*_2_ = (1,2). If job *J*_2_ = (1,2) is scheduled on the first machine, and there is no job arriving after, we have *C*_*A*_/*C*_opt_ = 2 > 3/2. Otherwise, job *J*_2_ is scheduled on the second machine, and we generate job *J*_3_ = (2,2). No matter which machine that job *J*_3_ is scheduled on, we have *C*_*A*_ = 3. Since the optimal algorithm will scheduled job *J*_1_ and job *J*_2_ on the first machine and scheduled job *J*_3_ on the second machine. Hence, *C*_*A*_/*C*_opt_ = 3/2. The proof is completed.

### 3.2. Optimal Semi-Online Algorithm *GoS-TH*

Since we know *T*_1_ in advance and all the jobs with *g*_*j*_ = 1 must be scheduled on the first machine, we can regard them as one job; that is, *J*_0_ = (*T*_1_, 1). We scheduled job *J*_0_ on the first machine at first and do not need to care about the job with *g*_*j*_ = 1 later.

At the arrival of each job, *H* is updated to become a half of the total processing time of the jobs which include the jobs with *GoS* = 2 and job *J*_0_;  *P* is updated to become the maximum processing time. We define *P* ^*j*^, *S*_1_^*j*^, *S*_2_^*j*^, and *H* ^*j*^ to be *P*, *S*_1_, *S*_2_, and *H* after we scheduled job *J*_*j*_. Then, clearly the optimum makespan *C*_opt_ ≥ *L* = max⁡(*T*, *P*). Combined with the online algorithm presented by Park et al. [8], we propose Algorithm *GoS*-*TH*.

*Algorithm  GoS-TH*

1. Scheduled *J*_0_ to the first machine and *t*(*S*_1_^0^) = *T*_1_.
2. Let *p*_*j*_ = 0 for all the jobs with *g*_*j*_ = 1.
3. Suppose that the incoming job is *J*_*j*_ and *g*_*j*_ = 1; assign job *J*_*j*_ to the first machine.
4. Suppose the incoming job is *J*_*j*_ and *g*_*j*_ = 2. *H*_*j*_ = *H*_*j*−1_ + *p*_*j*_/2, *P*_*j*_ = max⁡(*P*_*j*−1_, *p*_*j*_), and *L*_*j*_ = max⁡(*T*_*j*_, *P*_*j*_).
  (4.1) If *t*(*S*_2_^*j*^) + *p*_*j*_ ≤ (3/2)*L*_*j*_, scheduled it to the second machine.
  (4.2) If *t*(*S*_2_^*j*^) + *p*_*j*_ > (3/2)*L*_*j*_, scheduled it to the first machine.

**Theorem 2**

The competitive ratio of Algorithm *GoS-TH* is 3/2 for *P*2 · *GoS* | *sum* · *higher* | *C*_max⁡_.

**Proof**

Suppose that Theorem 2 is false. There must exist an instance with the least number of jobs to make *C*_*GoS*-*TH*_ > (3/2)*C*_opt_. The makespan is not determined until the arrival of job *J*_*n*_. Therefore,  

(1) $$\begin{array}{c} C_{GoS\text{-}TH}=\max\left\{t\left(S_{1}^{n}\right),t\left(S_{2}^{n}\right)\right\}>\frac{3}{2}C_{\text{opt}},\\ \max\left\{t\left(S_{1}^{n-1}\right),t\left(S_{2}^{n-1}\right)\right\}\leq \frac{3}{2}C_{\text{opt}}. \end{array}$$

If *g*_*n*_ = 1, based on Algorithm *GoS*-*TH*, we have *t*(*S*_1_^*n*^) = *t*(*S*_1_^*n*−1^) and *t*(*S*_2_^*n*^) = *t*(*S*_2_^*n*−1^). Due to the fact that max⁡{*t*(*S*_1_^*n*−1^), *t*(*S*_2_^*n*−1^)} ≤ (3/2)*C*_opt_, we just need to prove Theorem 2 is true when *g*_*n*_ = 2.

If *g*_*n*_ = 2, when it is scheduled on the second machine, we have *C*_*GoS*-*TH*_ = *t*(*S*_2_^*n*^) = *t*(*S*_2_^*n*−1^) + *p*_*n*_ ≤ (3/2)*C*_opt_. This is contradicting with inequality (1). Otherwise, job *J*_*n*_ is scheduled on the first machine, which leads to *t*(*S*_2_^*n*−1^) + *p*_*n*_ > (3/2)*L*_*n*_ ≥ (3/2)*H*_*n*_; combined with (*t*(*S*_2_^*n*−1^) + *p*_*n*_ + *t*(*S*_1_^*n*−1^))/2 = *H*_*n*_, we get that *t*(*S*_1_^*n*−1^) < (1/2)*L*_*n*_ ≤ (1/2)*C*_opt_. Since *p*_*n*_ ≤ *C*_opt_, we have *t*(*S*_1_^*n*−1^) + *p*_*n*_ < (3/2)*C*_opt_, which also contradicted with inequality (1). The proof is completed.

## 4. Optimal Algorithm for *P*2 · *GoS* | *sum* · *lower* | *C*_max⁡_

In this section, we prove a lower bound of competitive ratio and present an optimal algorithm for the semi-online version as *T*_2_ is known in advance.

### 4.1. Lower Bound of Competitive Ratio

**Theorem 3**

Any semi-online algorithm for *P*2 · *GoS* | *sum* · *lower* | *C*_max⁡_ has a competitive ratio of at least 20/13.

**Proof**

We will construct a job sequence with *T*_2_ = 26 to make an arbitrary algorithm *A* behave poorly. We begin with jobs *J*_1_ = (1,2) and *J*_2_ = (1,2). We discuss the following three cases.

*Case* *1* (*J*_1_ and *J*_2_ are scheduled on the first machine). We continue to generate jobs *J*_3_ = (12,2) and *J*_4_ = (12,2). If job *J*_3_ and job *J*_4_ are scheduled on the second machine, we have *t*(*S*_2_) = 24; thus, *C*_*A*_/*C*_opt_ = 24/14 > 20/13. Otherwise, if job *J*_3_ or job *J*_4_ is scheduled on the first machine or both of them are scheduled on the first machine, we further generate job *J*_5_ = (26,1). We will have *t*(*S*_1_) ≥ 40. Since optimal algorithm will scheduled jobs *J*_1_, *J*_2_, *J*_3_, and *J*_4_ on the second machine and scheduled job *J*_5_ on the first machine, we have *C*_*A*_/*C*_opt_ ≥ 20/13.

*Case  2* (*J*_1_ or *J*_2_ is scheduled on the first machine). We continue to generate job *J*_3_ = (2,2). Then we discuss the following two subcases.

*Subcase* *2.1* (job *J*_3_ is scheduled on the first machine). We continue to generate jobs *J*_4_ = (11,2) and *J*_5_ = (11,2). If job *J*_4_ and job *J*_5_ are scheduled on the second machine, we have *t*(*S*_2_) = 23 and *t*(*S*_1_) = 2, which lead to *C*_*A*_/*C*_opt_ = 23/13 > 20/13. Otherwise, if job *J*_4_ or job *J*_5_ is scheduled on the first machine or both of them are scheduled on the first machine, we further generate job *J*_6_ = (26,1) which leads to *C*_*A*_/*C*_opt_ ≥ 20/13.

*Subcase* *2.2* (*J*_3_ is scheduled on the second machine). We generate job *J*_4_ = (5,2). If job *J*_4_ is scheduled on the second machine, we generate job *J*_5_ = (13,2). If job *J*_5_ is scheduled on the second machine, we have *t*(*S*_2_) = 21. Since the optimal algorithm will scheduled jobs *J*_1_, *J*_2_, *J*_3_, and *J*_4_ on the first machine and scheduled job *J*_5_ on the second machine, we have *C*_*A*_/*C*_opt_ = 21/13 > 20/13. If job *J*_5_ is scheduled on the first machine, we generate job *J*_6_ = (26,1) which leads to *C*_*A*_/*C*_opt_ ≥ 20/13.

Otherwise, if job *J*_4_ is scheduled on the first machine, we generate job *J*_5_ = (8,2). If job *J*_5_ is scheduled on the first machine, we generate job *J*_6_ = (26,1) which leads to *C*_*A*_/*C*_opt_ ≥ 20/13. If job *J*_5_ is scheduled on the second machine, we generate job *J*_6_ = (9,2). If job *J*_6_ is scheduled on the second machine, we have *t*(*S*_2_) = 20 and *C*_opt_ = 13; thus, *C*_*A*_/*C*_opt_ = 20/13. If *J*_6_ is scheduled on the first machine, we generate job *J*_7_ = (26,1) and have *t*(*S*_1_) = 40 and *C*_opt_ = 26, which also lead to *C*_*A*_/*C*_opt_ = 20/13.

*Case* *3* (*J*_1_ and *J*_2_ are scheduled on the second machine). We continue to generate job *J*_3_ = (2,2). Then we discuss the following two subcases.

*Subcase* *3.1* (job *J*_3_ is scheduled on the second machine). We generate jobs *J*_4_ = (3,2) and *J*_5_ = (3,2). If job *J*_4_ or job *J*_5_ is scheduled on the second machine or both of them are scheduled on the second machine, we further generate job *J*_6_ = (1,2). If job *J*_6_ is scheduled on the second machine, we generate job *J*_7_ = (14,2). If job *J*_7_ is scheduled on the second machine, we have *t*(*S*_2_) = 22. Since the optimal algorithm will scheduled jobs *J*_1_, *J*_2_, *J*_3_, *J*_4_, *J*_5_, and *J*_6_ on the first machine and scheduled job *J*_7_ on the second machine, we have *C*_*A*_/*C*_opt_ ≥ 22/14 > 20/13. Otherwise, job *J*_7_ is scheduled on the first machine, and then we generate job *J*_8_ = (26,1) and have *t*(*S*_1_) ≥ 40 and *C*_opt_ = 26; hence, *C*_*A*_/*C*_opt_ > 20/13. If job *J*_6_ is scheduled on the first machine, we generate job *J*_7_ = (13,2). If job *J*_7_ is scheduled on the second machine, we have *t*(*S*_2_) ≥ 20 and *C*_opt_ = 13, hence, we also have *C*_*A*_/*C*_opt_ > 20/13. Otherwise, job *J*_7_ is scheduled on the first machine, and then we generate job *J*_8_ = (26,1). We have *C*_*A*_/*C*_opt_ > 20/13.

If job *J*_4_ and job *J*_5_ are scheduled on the first machine, we generate jobs *J*_6_ = (8,2) and *J*_7_ = (8,2). If job *J*_6_ or job *J*_7_ is scheduled on the first machine or both of them are scheduled on the first machine, we further generate job *J*_8_ = (26,1). We have *C*_*A*_/*C*_opt_ > 20/13. Otherwise, if job *J*_6_ and job *J*_7_ are scheduled on the second machine, we have *t*(*S*_2_) = 20 and *C*_opt_ = 13, which lead to *C*_*A*_/*C*_opt_ = 20/13.

*Subcase* *3.2* (job *J*_3_ is scheduled on the first machine). We generate job *J*_4_ = (2,2). If job *J*_4_ is scheduled on the first machine, we generate jobs *J*_5_ = (10,2) and *J*_6_ = (10,2). If job *J*_5_ and job *J*_6_ are scheduled on the second machine, we have *t*(*S*_2_) = 22 and *C*_opt_ = 13; thus, *C*_*A*_/*C*_opt_ = 22/13 > 20/13. Otherwise, if job *J*_5_ or job *J*_6_ is scheduled on the first machine or both of them are scheduled on the first machine, we further generate job *J*_7_ = (26,1) and have *t*(*S*_1_) ≥ 40; thus, *C*_*A*_/*C*_opt_ = 20/13.

Otherwise, if job *J*_4_ is scheduled on the second machine, we generate job *J*_5_ = (4,2). If job *J*_5_ is scheduled on the second machine, we generate job *J*_6_ = (13,2). If job *J*_6_ is scheduled on the second machine, we have *t*(*S*_2_) = 21 and *C*_opt_ = 13; hence, *C*_*A*_/*C*_opt_ = 20/13. If job *J*_6_ is scheduled on the first machine, we generate job *J*_7_ = (26,1) and have *t*(*S*_1_) ≥ 41; thus, *C*_*A*_/*C*_opt_ = 41/26 > 20/13.

Therefore, job *J*_5_ is scheduled on the first machine. Then we generate jobs *J*_6_ = (8,2) and *J*_7_ = (8,2). If job *J*_6_ or job *J*_7_ is scheduled on the first machine or both of them are scheduled on the first machine, we further generate job *J*_8_ = (26,1). We will have *t*(*S*_1_) ≥ 40 and *C*_opt_ = 26; hence, *C*_*A*_/*C*_opt_ > 20/13. Otherwise, if job *J*_6_ and job *J*_7_ are scheduled on the second machine, we have *t*(*S*_2_) = 20 and *C*_opt_ = 13; also, we have *C*_*A*_/*C*_opt_ = 20/13. The proof is completed.

### 4.2. Optimal Semi-Online Algorithm *GoS-TL*

In this subsection, we design an optimal algorithm with a competitive ratio of 20/13. Let *x*_1_ and *x*_2_ be the jobs with *g*_*j*_ = 2 assigned to the first and second machine, respectively, where *t*(*x*_1_) + *t*(*x*_2_) = *T*_2_. We define *x*_1_^*j*^ and *x*_2_^*j*^ to be *x*_1_ and *x*_2_ after we scheduled job *J*_*j*_. Then, we propose Algorithm *GoS*-*TL*.

*Algorithm  GoS-TL*

1. Suppose that the incoming job is *J*_*j*_ and *g*_*j*_ = 1; assign job *J*_*j*_ to the first machine.
2. Suppose that the incoming job is *J*_*j*_ and *g*_*j*_ = 2.
  (2.1) (*Stopping criterion 1*). If (3/13)*T*_2_ ≤ *t*(*x*_1_^*j*−1^) + *p*_*j*_ ≤ (7/13)*T*_2_, scheduled job *J*_*j*_ and all the remaining jobs with *GoS* = 1 to the first machine, and then scheduled all the remaining jobs with *GoS* = 2 to the second machine. Stop.
  (2.2) (*Stopping criterion 2*). If (6/13)*T*_2_ ≤ *t*(*x*_2_^*j*−1^) + *p*_*j*_ ≤ (10/13)*T*_2_, scheduled job *J*_*j*_ to the second machine and scheduled all the remaining jobs to the first machine. Stop.
  (2.3) (*Stopping criterion 3*). If *t*(*x*_2_^*j*−1^) > 10/13, scheduled job *J*_*j*_ and the remaining jobs to the first machine. Stop.
  (2.4) If *t*(*x*_2_^*j*−1^) ≤ (7/26)*T*_2_ and (7/13)*T*_2_ ≤ *t*(*x*_2_^*j*−1^) + *p*_*j*_ < (6/13)*T*_2_, scheduled job *J*_*j*_ to the first machine. Continue.
  (2.5) Otherwise, scheduled job *J*_*j*_ to the second machine. Continue.

**Lemma 4**

If (6/13)*T*_2_ ≤ *t*(*x*_2_) ≤ (10/13)*T*_2_, then *C*_*GoS*-*TL*_ ≤ (20/13)*C*_*opt*_.

**Proof**

Since *C*_opt_ ≥ (1/2)*T*_2_, we have *t*(*S*_2_) = *t*(*x*_2_) ≤ (10/13)*T*_2_ ≤ (20/13)*C*_opt_. Then, we only need to prove that *t*(*S*_1_) ≤ (20/13)*C*_opt_. We discuss it by the following two cases.

*Case* *1* (*T*_1_ ≥ *T*_2_). In this case, we have *t*(*S*_1_) = *t*(*x*_1_) + *T*_1_ and *T*_1_ ≥ (*T*_1_ + *T*_2_)/2. Since (6/13)*T*_2_ ≤ *t*(*x*_2_) ≤ (10/13)*T*_2_, we have *t*(*x*_1_) ≤ (7/13)*T*_2_. Combined with *C*_opt_ ≥ *T*_1_, we have

(2) $$\begin{array}{c} \frac{C_{GoS\text{-}TL}}{C_{\text{opt}}}\leq \frac{\left(7/13\right)T_{2}+T_{1}}{T_{1}}\leq \frac{\left(7/13\right)T_{1}+T_{1}}{T_{1}}=\frac{20}{13}. \end{array}$$

*Case* *2* (*T*_1_ < *T*_2_). In this case, we have *T*_1_ < (*T*_1_ + *T*_2_)/2, which leads to *C*_opt_ ≥ (*T*_1_ + *T*_2_)/2. Then, we have

(3) $$\begin{array}{c} \frac{C_{GoS\text{-}TL}}{C_{\text{opt}}}\leq \frac{\left(14/13\right)T_{2}+2T_{1}}{T_{1}+T_{2}}. \end{array}$$

Since ((14/13)*T*_2_ + 2*T*_1_)/(*T*_1_ + *T*_2_) is increasing function of the variation of *T*_1_, thus, we have

(4) $$\begin{array}{c} \frac{C_{GoS\text{-}TL}}{C_{\text{opt}}}\leq \frac{\left(14/13\right)T_{2}+2T_{1}}{T_{1}+T_{2}}<\frac{\left(14/13\right)T_{2}+2T_{2}}{T_{2}+T_{2}}=\frac{20}{13}. \end{array}$$

The proof is completed.

Based on Lemma 4, we straightforwardly have Corollary 5.

**Corollary 5**

(1) If *t*(*x*_1_) ≤ (7/13)*T*_2_, then *t*(*S*_1_) ≤ (20/13)*C*_*opt*_; (2) if *t*(*x*_2_) ≤ (10/13)*T*_2_, then *t*(*S*_2_) ≤ (20/13)*C*_*opt*_.

**Lemma 6**

*t*(*x*_1_) ≤ (7/13)*T*_2_.

**Proof**

Since Algorithm *GoS*-*TL* will only scheduled a job with *g*_*j*_ = 2 to the first machine only when *t*(*x*_1_^*j*−1^) + *p*_*j*_ ≤ (7/13)*T*_2_, the lemma can directly be got from the Algorithm *GoS*-*TL*. The proof is completed.

By using Corollary 5 and Lemma 6, we can obtain the following corollary.

**Corollary 7**

If *t*(*x*_2_) ≤ (10/13)*T*_2_, then *C*_*GoS*-*TL*_ ≤ (20/13)*C*_*opt*_.

Based on Lemma 6 and Corollary 7, if we prove *t*(*S*_2_) ≤ (20/13)*C*_opt_ will hold when *t*(*x*_2_) > (10/13)*T*_2_, then we can prove that Algorithm *GoS*-*TL* is (20/13)-competitive.

**Lemma 8**

If job *J*_*j*_ is scheduled on the second machine by Algorithm *GoS*-*TL* where *t*(*x*_2_^*j*−1^) + *p*_*j*_ > (10/13)*T*_2_ and *t*(*x*_2_^*j*−1^)≤(7/26)*T*_2_, then *t*(*S*_2_)<(20/13)*C*_*opt*_.

**Proof**

If *t*(*x*_2_^*j*−1^) + *p*_*j*_ > (10/13)*T*_2_ and *t*(*x*_2_^*j*−1^) ≤ (7/13)*T*_2_, we have *p*_*j*_ > (1/2)*T*_2_. Then we have *C*_opt_ ≥ *p*_*j*_ > (1/2)*T*_2_. If job *J*_*j*_ is scheduled on the second machine, Algorithm *GoS*-*TL* will scheduled the remaining jobs on the first machine. Thus,

(5) $$\begin{array}{c} \frac{t\left(S_{2}^{j}\right)}{C_{\text{opt}}}\leq \frac{t\left(S_{2}^{j}\right)}{p_{j}}=\frac{t\left(x_{2}^{j-1}\right)+p_{j}}{p_{j}}. \end{array}$$

Since (*t*(*x*_2_^*j*−1^) + *p*_*j*_)/*p*_*j*_ is decreasing function of the variation of *p*_*j*_ and increasing function of variation of *t*(*x*_2_^*j*−1^), we have

(6) $$\begin{array}{c} \frac{t\left(S_{2}^{j}\right)}{C_{\text{opt}}}\leq \frac{t\left(x_{2}^{j-1}\right)+p_{j}}{p_{j}}<\frac{\left(7/26\right)T_{2}+\left(1/2\right)T_{2}}{\left(1/2\right)T_{2}}=\frac{20}{13}. \end{array}$$

The proof is completed.

**Lemma 9**

If (6/13)*T*_2_ ≤ *t*(*x*_2_^*j*−1^) ≤ (10/13)*T*_2_, then *t*(*S*_2_) > (10/13)*T*_2_ will never happen.

**Proof**

If (6/13)*T*_2_ ≤ *t*(*x*_2_^*j*−1^)≤(10/13)*T*_2_, Algorithm *GoS*-*TL* will scheduled the remaining jobs on the first machine, so *t*(*S*_2_) = *t*(*x*_2_^*j*−1^)≤(10/13)*T*_2_. The proof is completed.

**Lemma 10**

If job *J*_*j*_ is scheduled on the second machine by Algorithm *GoS*-*TL* where (7/26)*T*_2_ < *t*(*x*_2_^*j*−1^)<(6/13)*T*_2_ and *t*(*x*_2_^*j*^)>(10/13)*T*_2_, then *t*(*S*_2_) = *t*(*x*_2_^*j*^)<(20/13)*C*_*opt*_.

**Proof**

Let *J*_*w*_ be the job that is scheduled on the second machine by Algorithm *GoS*-*TL* which satisfies *t*(*x*_2_^*w*−1^) ≤ 7/13 and (7/26)*T*_2_ < *t*(*x*_2_^*w*−1^) + *p*_*w*_ < (6/13)*T*_2_. Since *J*_*w*_ is scheduled on the second machine by Algorithm *GoS*-*TL* and (7/26)*T*_2_ < *t*(*x*_2_^*w*−1^) + *p*_*w*_ < (6/13)*T*_2_, we have *t*(*x*_1_^*w*−1^) + *p*_*w*_ > (7/13)*T*_2_; otherwise, Algorithm *GoS*-*TL* will scheduled job *J*_*w*_ on the first machine. Moreover, *t*(*x*_1_^*w*−1^) < (3/13)*T*_2_ must hold which leads to *p*_*w*_ > (4/13)*T*_2_. This further implies that *t*(*x*_2_^*w*−1^) < (2/13)*T*_2_ since *t*(*x*_2_^*w*−1^) + *p*_*w*_ < (6/13)*T*_2_.

If *t*(*x*_1_^*w*−1^) = 0, then *p*_*w*_ > (7/13)*T*_2_ which leads to *t*(*x*_2_^*w*^) > (6/13)*T*_2_; this is contradicting with the definition of job *J*_*w*_. Therefore, we have *t*(*x*_1_^*w*−1^) > 0. Based on step (2.4) of Algorithm *GoS*-*TL*, the processing time of the job that is assigned to *x*_1_^*w*−1^ is larger than (3/26)*T*_2_ since *t*(*x*_2_^*w*−1^) < (2/13)*T*_2_. Combined with *t*(*x*_1_^*w*−1^) < (3/13)*T*_2_, we know there is only one job in *x*_1_^*w*−1^, call it job *J*_*v*_. Since job *J*_*v*_, job *J*_*w*_, and *t*(*x*_2_^*w*−1^) need to satisfy

(7) $$\begin{array}{c} p_{v}+p_{w}>\frac{7}{13}T_{2},\\ p_{v}+t\left(x_{2}^{w-1}\right)>\frac{7}{26}T_{2},\\ \frac{7}{26}T_{2}<t\left(x_{2}^{w-1}\right)+p_{w}<\frac{6}{13}T_{2}, \end{array}$$

we can get *p*_*v*_ > (9/52)*T*_2_, which implies that *t*(*S*_2_) < (43/52)*T*_2_.

Since there is no job with *g*_*j*_ = 2 that will scheduled on the first machine between job *J*_*v*_ and *J*_*j*_ by Algorithm *GoS*-*TL*, combined with job *J*_*j*_ being scheduled on the second machine, we have *p*_*v*_ + *p*_*j*_ > (7/13)*T*_2_. Since *p*_*j*_ > (4/13)*T*_2_, *p*_*v*_, *p*_*w*_, and *p*_*j*_ must satisfy

(8) $$\begin{array}{c} p_{v}+p_{w}>\frac{7}{13}T_{2},\\ p_{v}+p_{j}>\frac{7}{13}T_{2},\\ p_{w}+p_{j}\geq \frac{9}{13}T_{2}, \end{array}$$

which implies that *C*_opt_ > (7/13)*T*_2_, hence,

(9) $$\begin{array}{c} \frac{t\left(S_{2}\right)}{C_{\text{opt}}}<\frac{\left(43/52\right)T_{2}}{\left(7/13\right)T_{2}}=\frac{20}{13}. \end{array}$$

The proof is completed.

Based on Lemma 4 to Lemma 10 and Corollary 5 to Corollary 7, we have the following theorem naturally.

**Theorem 11**

The competitive ratio of Algorithm *GoS-TL* is 20/13 for *P*2 · *GoS* | *sum* · *lower* | *C*_max⁡_.

## 5. Optimal Algorithm for *P*2 · *GoS* | *sum* · *high*&*sum* · *lower* | *C*_*max*_

In this section, we show a lower bound of competitive ratio and present an optimal algorithm for the semi-online version as *T*_1_ and *T*_2_ are known in advance.

### 5.1. Lower Bound of Competitive Ratio

**Theorem 12**

Any semi-online algorithm for *P*2 · *GoS* | *sum* · *high*&*sum* · *lower* | *C*_max⁡_ has a competitive ratio of at least 4/3.

**Proof**

The theorem will be proved by adversary method. Let *T*_1_ = 1/3 and *T*_2_ = 5/3 be known in advance. The first job is *J*_1_ = (1/3, 1). Job *J*_1_ must be scheduled on the first machine. Then job *J*_2_ = (1/3, 2) arrives. If job *J*_2_ is scheduled on the second machine, we further generate jobs *J*_3_ = (1,2) and *J*_4_ = (1/3, 2). In this situation, we have *C*_*A*_ ≥ 4/3 and *C*_opt_ = 1 since the optimal algorithm will scheduled jobs *J*_1_, *J*_2_, and *J*_4_ on the first machine and scheduled job *J*_3_ on the second machine. Thus, we have *C*_*A*_/*C*_opt_ ≥ 4/3. Otherwise, if job *J*_2_ is scheduled on the first machine, then we further generate jobs *J*_3_ = (2/3, 2) and *J*_4_ = (2/3, 2). In this situation, we also have *C*_*A*_ ≥ 4/3 and *C*_opt_ = 1 since the optimal algorithm will scheduled jobs *J*_1_ and *J*_3_ on the first machine and scheduled jobs *J*_2_ and *J*_3_ on the second machine. The proof is completed.

### 5.2. Optimal Semi-Online Algorithm *GoS-TB*

In this subsection, we design an optimal algorithm with a competitive ratio of 4/3. Since we know *T*_1_ in advance and all the jobs with *g*_*j*_ = 1 must be scheduled on the first machine, therefore, we can regard them as one job, that is, *J*_0_ = (*T*_1_, 1). We scheduled job *J*_0_ on the first machine at first and do not need to care about the job with *g*_*j*_ = 1 later. We present Algorithm *GoS*-*TB* as follows.

*Algorithm GoS-TB*

1. Scheduled *J*_0_ to the first machine and *t*(*S*_1_^0^) = *T*_1_.
2. Let *p*_*j*_ = 0 for all the jobs with *g*_*j*_ = 1.
3. Suppose that the incoming job is *J*_*j*_ and *g*_*j*_ = 1; assign job *J*_*j*_ to the first machine.
4. Suppose that the incoming job is *J*_*j*_ and *g*_*j*_ = 2.
  (4.1) If *t*(*S*_1_) + *p*_*j*_ ≤ (2/3)(*T*_1_ + *T*_2_), scheduled it to the first machine.
  (4.2) (*Stopping criterion*). Suppose *t*(*S*_1_) + *p*_*j*_ > (2/3)(*T*_1_ + *T*_2_).
    (4.2.1) If *t*(*S*_1_^*j*−1^) > (1/3)(*T*_1_ + *T*_2_), scheduled job *J*_*j*_ and the remaining jobs with *GoS* = 2 on the second machine and scheduled the remaining jobs with *GoS* = 1 to the first machine. Stop.
    (4.2.2) If *t*(*S*_1_^*j*−1^) ≤ (1/3)(*T*_1_ + *T*_2_), scheduled job *J*_*j*_ to the second machine, and scheduled the remaining jobs to the first machine. Stop.

**Theorem 13**

The competitive ratio of Algorithm *GoS*-*TH* is 4/3 for *P*2 · *GoS* | *sum* · *high*&*sum* · *lower* | *C*_max⁡_.

**Proof**

Since the job with *g*_*j*_ = 1 is scheduled at first and we do not need to care about them after that. We focus on the jobs with *g*_*j*_ = 2.

Assume job *J*_*j*_ is the first job with *g*_*j*_ = 2 to make *t*(*S*_1_) + *p*_*j*_ > (2/3)(*T*_1_ + *T*_2_). We prove it by the following two cases.

*Case* *1* (*t*(*S*_1_^*j*−1^)>(1/3)(*T*_1_ + *T*_2_)). In this case, Algorithm *GoS*-*TB* schedules job *J*_*j*_ and the remaining jobs with *GoS* = 2 on the second machine. Therefore, *t*(*S*_1_) ≤ (2/3)(*T*_1_ + *T*_2_) and *t*(*S*_2_) ≤ *T*_1_ + *T*_2_ − *t*(*S*_1_) < (2/3)(*T*_1_ + *T*_2_). Since *C*_opt_ ≥ (1/2)(*T*_1_ + *T*_2_), we have *C*_*GoS*-*TH*_ = max⁡{*t*(*S*_1_), *t*(*S*_2_)} ≤ (2/3)(*T*_1_ + *T*_2_) ≤ (4/3)*C*_opt_.

*Case* *2* (*t*(*S*_1_^*j*−1^)≤(1/3)(*T*_1_ + *T*_2_)). In this case, we have *p*_*j*_ > (1/3)(*T*_1_ + *T*_2_). Algorithm *GoS*-*TH* will only scheduled job *J*_*j*_ on the second machine and scheduled the remaining jobs on the first machine. If *p*_*j*_ ≥ (1/2)(*T*_1_ + *T*_2_), we have *C*_opt_ = *p*_*j*_ and *t*(*S*_1_) ≤ *p*_*j*_ = *t*(*S*_2_). Therefore, *C*_*GoS*-*TH*_ = *C*_opt_. Otherwise, (1/3)(*T*_1_ + *T*_2_) < *p*_*j*_ < (1/2)(*T*_1_ + *T*_2_), which leads to *t*(*S*_1_) ≤ *T*_1_ + *T*_2_ − *t*(*S*_2_) < (2/3)(*T*_1_ + *T*_2_). Hence, we have *C*_*GoS*-*TH*_ = max⁡{*t*(*S*_1_), *t*(*S*_2_)} ≤ (2/3)(*T*_1_ + *T*_2_) ≤ (4/3)*C*_opt_. The proof is completed.
